# Application of time-series regularity metrics to ion flux data from a population of pollen tubes

**DOI:** 10.1080/19420889.2021.1899574

**Published:** 2021-03-19

**Authors:** Mariusz A. Pietruszka

**Affiliations:** Faculty of Natural Sciences, Institute of Biology, Biotechnology and Environmental Protection, University of Silesia, Katowice, Poland

**Keywords:** Collective excitations, critical temperature, emergent behavior, Hurst exponent, Kolmogorov-Sinai entropy, Lyapunov exponent, optimum growth, temperature coding, tobacco

## Abstract

Detecting the presence of an irregularity/regularity or chaos in the ion flows of an evolving plant cell is an important task that can be unraveled by performing the analyses by different metrics. Here I show that the results of the advanced fluctuation estimation methods that are obtained from the time series that is generated by the extracellular ion fluxes of tobacco pollen tubes (*Nicotiana tabacum* L.) have long-range correlations at critical temperatures. Further experimental evidence has been found to support the claim that the autonomous growth organization of extreme plant cell expansion is accomplished by self-organizing criticality (SOC), which is an orchestrated instability that occurs in an optimally evolving cell. The temperature-induced synchronous action of the ionic fluxes that are manifested, *inter alia*, by minimal dynamic entropy enabled the *molecularly encoded* information about germination and optimal growth temperatures of tobacco pollen tubes to be determined.

In seed plants, the pollen tube is a cellular extension that serves as a conduit through which the male gametes pass until the egg is fertilized. It consists of a single elongated cell that has a distinctive feature that changes its growth rate periodically [e.g., 1]. Pollen tubes have an extremely rapid growth rate that can also be reproduced *ex vivo*. They are highly polarized tip-growing cells that depend on the cytosolic pH gradients for signaling and growth [2]. Plasma membrane H^+^-ATPase has been theoretically proposed to supply the energy for pollen tube growth and underlie, throughout the chemical potential of H^+^ ions, the synchronous growth oscillations [3]. These predicted pH/growth rate cross-correlations have recently been confirmed empirically [2] with pH having crucial roles in regulating pollen tube growth.

In our previous work [4], among others, the bioelectric behavior of tobacco pollen tubes (*Nicotiana tabacum* L.) was examined. It was shown that the scale-free processes that result from the critical phenomena can be an essential property of a living cell. In particular, the canonical value of the spectral exponent ($\beta_{c}=1$), determined by the slope of the power spectral density (PSD) function, was obtained for the so-called flicker noise (pink) at the optimal growth temperature. However, the spectral exponent $\left(\beta \right)$ was the only measure (quantity) that was specified for the entire range of (physiological) temperatures concerned and therefore it should be supplemented with other statistical metrics.

Here, I evaluated quantitatively (numerically) the advanced statistical measures, namely the Hurst exponent, the largest (maximal) Lyapunov exponent (LLE) and the Kolmogorov-Sinai dynamic entropy of an experimental time series for the detected external ion fluxes from elongating pollen tubes. I also reconstructed the corresponding phase space according to Takens’ theorem.

The Hurst exponent [5] is used to measure the long-term memory of a time series. It refers to the autocorrelation of a time series and the rate at which it decreases with increasing delay between the pairs of values [6]. The Lyapunov exponent is by definition the rate of the exponential separation with time of the initially close trajectories. It describes the speed of the convergence or divergence of the trajectories in each dimension of the attractor and estimates the amount of chaos in a system [7,8]. Dynamic entropy [9] quantifies the size of the fluctuation regularity in a time series. A low entropy value indicates that a time series is deterministic, while a high value indicates its randomness [10,11]. All of these measures, which enable the level of irregularity/chaos to be estimated, were compared with our recent results for calculating the spectral exponent of the linearly approximated PSD for the same experimental data (time series).

## Signatures of coherent dynamics in the extracellular ionic fluxes of pollen tubes

Entropy is a crucial state variable in thermodynamics, statistical mechanics, quantum mechanics and information processing [9] that carries global information about whether a system is ordered, less ordered or disordered. The dynamic entropy of a living system (ensemble of pollen tubes), which is considered in this article, is *not* a function of the state of a system, but a function of its dynamics. It was found that the dynamic entropy of extracellular ionic fluxes of tobacco pollen tubes (net current) as a function of temperature can be calculated by analyzing the time series of the electromotive force (total voltage) that is generated by the unperturbed ion fluxes. In the case of strong correlations, due to critical fluctuations, the dynamic entropy of the tobacco pollen tubes has a minimum at the characteristic temperatures for germination and optimal growth.

**Figure 1.**
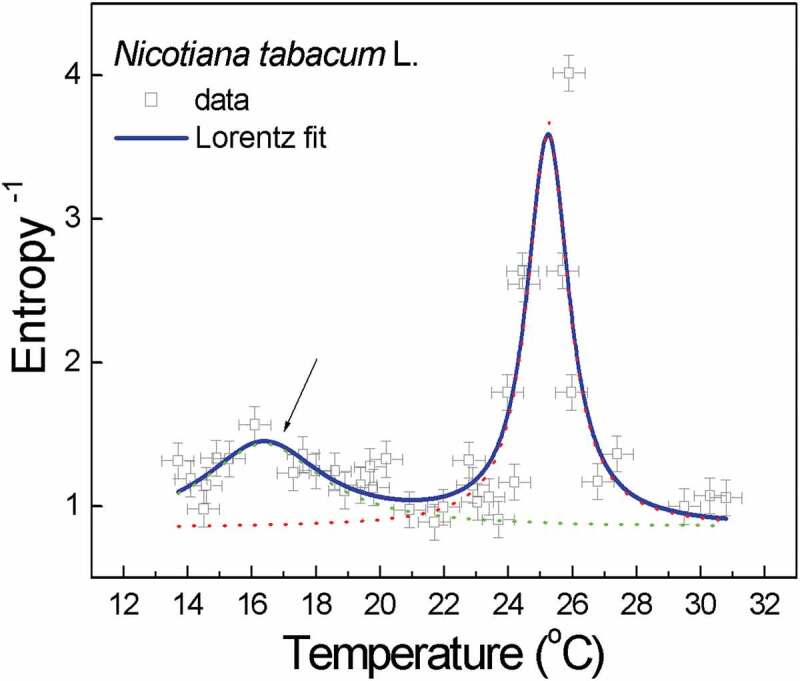
Double resonance curve of the reciprocal of the calculated (approximate) dynamic entropy of the extracellular ion fluxes as a function of temperature for the pollen tubes of *Nicotiana tabacum* L. The determined germination temperature was *T_c_^ger^* = 16.37 ± 0.24°C, while for the optimal growth, it was *T_c_^opt^* = 25.25 ± 0.03°C. The solid royal blue line corresponds to the multi-peak Lorentz fit (*R*^2^ = 0.69), which is interpolated by the B-spline. The errors (empirical: *x_err_* = ± 0.5°C; from Lorentz fit: *y_err_* = ± 0.125) are represented by the drone-like objects in the plot. The arrow indicates the peak of reverse entropy (corresponding to the local minimum of dynamic entropy) that is associated with germination. The approximate entropy does reflect closely what was obtained for the spectral exponent before [compare with Fig. 5 in 4]

The results of the calculations are shown in Table 1 (to be on the safe side, I calculated the approximate ($S_{a}$) and the sample ($S_{s}$) entropy), which presents the representative values (from 43), and in Figure 1. The latter, however, deserves attention first. Note that in Figure 1, the gray points (which are *not* experimental points) result from the approximate entropy calculation [12]. Each one is derived from N = 5000 voltage time series (EMF) measurement points, which are generated as pink noise by the extracellular ion fluxes of elongating pollen tubes. After interpolation (Lorentz fit), the maximum values in the diagram indicate the characteristic (critical) temperatures in the tested system in accordance with the literature data. However, this likely means that both of these physiologically relevant temperatures (germination and optimal growth) have been fine-tuned and molecularly encoded. Otherwise, these characteristic temperatures would not be repeated in the next generations.

**Table 1.** *Nicotiana tabacum* L. pollen tubes. Analysis of the extracellular ion fluxes at a critical temperature and beyond – dynamic metrics

Temperature	Spectral signature (β)*	Corrected R/S Hurst exponent**	Largest Lyapunov exponent (emb. dims)	Approximate entropy ($S_{a}$)	Sample entropy ($S_{s}$)
25.9 ± 0.5°C (T = T_c_)	1.0067 ± 0.024	0.9696445	−15.85 (12)	0.2490113 Low value *Deterministic*	0.1622784
19.8 ± 0.5°C (T ≠ T_c_)	0.632 ± 0.025	0.6743406	−16.24 (12)	0.8849474 High value *Random*	0.7860483

*) Data from [4], showing the canonical case ($\frac{1}{f^{\beta }}$, β=1]) of pink (flicker) noise at the optimum temperature of 25.9 ± 0.5°C. Pink noise is a signal or process in the frequency spectrum of whose PSD is inversely proportional to frequency. The PSD of pink noise drops 10 dB per decade. The value of β at 19.8 ± 0.5°C though still at a pink noise range (in broad sense) is nearing the white noise, which is a random signal having equal intensity at different frequencies, giving it a constant PSD.

**) Calculated with the R [12] Practical Numerical Math Functions package. The hurstexp(*x*) function calculates the Hurst exponent of a time series *x* using R/S analysis [5,6].

While the Hurst exponent (Table 1) represents the expected quantities that are similar to those presented in 4, the negative LLE values presumably reflect the periodic or quasi-periodic (pH ?) oscillations that are observed in pollen tubes. As such, they may indeed reproduce a stable deterministic component that underlies the dynamics of this system. However, one must note that the series is non-stationary and violates the assumptions of the LLE calculations, which is fine since a completely conservative and periodic system would be in conflict with the flicker noise that is considered in the paper. Thus, while LLEs probably indicate a stable periodic component in the series, they do not provide a complete description of the dynamics, which was formerly determined by the spectral exponent.

This apparent inconsistency can be resolved by noting that the deterministic component in the signal (Table 1) comes from synchronized wave form [e.g., plasmon-polariton oscillations, 13], while the chaotic (random) component is the usual pink channel noise. In this context, the attractor reconstruction according to Takens’ theorem (not shown) was even more appealing. There, the states of the system that are constructed through the condensation in the lowest energy state – corresponding to the minimum entropy – of the quanta associated with the long-range correlations change the three-dimensional spherically symmetric-phase space outside the transition area into its two-dimensional projection, or axially elongated (quasi-one-dimensional) ellipsoid at the critical point. Hence, a spontaneous symmetry breaking that is reflected in the phase space occurs, which indicates a *dynamic phase transition* in the system with a change in the control parameter, i.e., temperature. The latter, however, presumably reflects the formation of the onset (like in the two-fluid model: condensate/non-condensate fraction) of the dynamic collective modes [14,15].

It seems that the obtained results can be framed in the research line that showed that macroscopic coherent states are formed in the processes of quantum condensation at the microscopic level [16] in nonequilibrium (dissipative) systems. Coherent states have (fractal) self-similarity properties, which have already been attributed to the scale-free dynamics of the critical spectral exponent ($\beta_{c}$) in [4]. However, as S approaches zero in $T_{c}$ (Figure 1), there are no (or insignificant) gradients with respect to temperature or chemical potential, but flux fluctuations are still there. Therefore, it seems appropriate to further describe this very specific activity of ions (the ionic avalanches [17], or super-diffusion/superfluid component) at the critical temperature in the formalism of dissipative systems [18].

A recurrent idea in the investigation of complex systems is that optimal (information) processing is to be found near phase transitions [19]. However, to my best knowledge, this assumption has no experimental realizations where a biologically relevant quantity is optimized at criticality. The presented results exemplify a network of excitable elements (*living* cells) at a critical point in non-equilibrium phase transition. Synchronization (avalanches) and already mentioned global oscillations may also emerge from the system dynamics. Needless to say, the synchronized ion avalanches (SOC) that are considered in this report may also be related to the microscopic explanation of the oscillating growth characteristics of the pollen tubes of tobacco [e.g., Fig. 6.1 and Fig. 6.3 in 1]. This issue, however, must undergo further in-depth analysis in future works.

The analysis according to five different indexes confirmed the previously observed criticality of the plant cell ensemble living under optimal temperature conditions and an excellent agreement with our previous findings for the spectral exponent was achieved in the calculated minimum for entropy. The observed long-range correlations, or even coherence, that occur at the optimal growth temperature, which are expressed by the lowest dynamic entropy value, indicate a synchronous (wave) operation without scattering (dispersion) of the ion/electron collective excitations, which presumably paves the way for innovative research in this emerging multidisciplinary field.

**Methods** The measurement system consisted of an external polystyrene thermostat and an internal Al-coated polystyrene measurement chamber containing a semiconductor-solute interface [ELoPvC detector, 20]. The sample containing the *Nicotiana tabacum* L. pollen tubes was placed in a liquid (conductive) germination medium [see 4, for details]. In the physiological temperature range, DC voltage measurements were taken (capturing a mean field of a collective of cells) at 4.1 Hz sampling with a DMM 4040 6–1/2 Digit Precision Multimeter from Tektronix, Inc. and then recorded as a 20 min. time series (N = 5000) on external media. The temperature control system consisted of an integrated control circuit and a 1 W heater (ceramic resistor) or ice added at temperatures below ambient temperature. The series of time data, which were collected at each temperature using this noninvasive solute–semiconductor interface technique, were detrended and analyzed by a program written by the author in R.
